# State of progress in treating cystic fibrosis respiratory disease

**DOI:** 10.1186/1741-7015-10-88

**Published:** 2012-08-10

**Authors:** Patrick A Flume, Donald R Van Devanter

**Affiliations:** 1 Medical University of South Carolina, Charleston, SC, USA; 2 Case Western Reserve University School of Medicine, Cleveland, OH, USA

**Keywords:** cystic fibrosis, pathophysiology, treatment

## Abstract

Since the discovery of the gene associated with cystic fibrosis (CF), there has been tremendous progress in the care of patients with this disease. New therapies have entered the market and are part of the standard treatment of patients with CF, and have been associated with marked improvement in survival. Now there are even more promising therapies directed at different components of the pathophysiology of this disease. In this review, our current knowledge of the pathophysiology of lung disease in patients with CF is described, along with the current treatment of CF lung disease, and the therapies in development that offer great promise to our patients.

## Introduction

Cystic fibrosis (CF) is a genetic disease resulting in complications in multiple organs, but especially involving the lungs and pancreas. CF is associated with considerable morbidity and an early demise, although the last two decades have realized a remarkable improvement in health and survival such that the median age of predicted survival now approaches 40 years [1]. This is the result of a combination of factors including the development of a sophisticated comprehensive care network and the use of several new medications developed specifically for the treatment of CF-related disease. This is a review of the current state of the treatment of CF lung disease and a look to the future development of promising treatment options.

## Pathophysiology of disease in cystic fibrosis

CF is an autosomal recessive disease resulting from the inheritance of a mutant allele of the gene for cystic fibrosis transmembrane conductance regulator (*CFTR*) from each parent [2-4]. CFTR is a c-AMP-regulated anion channel normally expressed on the apical surfaces of specialized epithelial cells, including those of the sweat glands, pancreas, and gastrointestinal and reproductive tracts, as well as airway epithelia and submucosal glands [5,6]. Although the transport of chloride ions across membranes is a recognized and critical CFTR function [6], there are other proposed CFTR-mediated effects, such as transport of bicarbonate [7]. Inheritance of mutant *CFTR *alleles is accompanied by a qualitative and/or quantitative reduction of CFTR activity at the cell surface; the extent to which CFTR activity is reduced influences the diversity and severity of pathophysiologic sequelae associated with CF [6]. Individuals carrying a single *CFTR *mutation may retain as little as 50% of wild-type CFTR activity but are unaffected. Persons with CF (that is, carrying two mutant *CFTR *alleles) in which one mutation retains residual (but reduced) CFTR function have less aggressive disease phenotypes and better overall survival than their peers who carry mutations in which very little or no CFTR activity is retained [6].

Pathophysiologic manifestations of CF arise in several organ systems *in utero*. Neonates with CF are identified for further screening evaluation in part based upon elevated levels of circulating immunoreactive trypsinogen caused by pancreatic ductile or duct blockage, pancreatic autodigestion, and leakage of digestive enzymes into the systemic circulation [8]. From birth, reduced CFTR activity in the sweat glands of people with CF hampers that individual's ability to recover salt from their sweat [6]. This provides the basis for the definitive test for CF: pilocarpine-induced iontophoresis (the CF 'sweat test'), with the majority of patients having chloride concentrations in sweat exceeding 60 mEq/L [8]. Reduced water and bicarbonate secretion in the gut may lead to meconium ileus in 10% to 15% of CF neonates [6]. Finally, most, if not all, males with CF are born without a palpable vas deferens [6].

**Figure 1:**
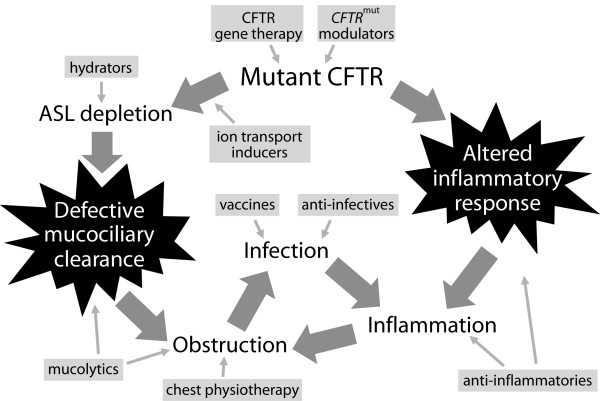
**Therapeutic targets of cystic fibrosis respiratory therapies**. Physiological ramifications of reduced *CFTR *activity in the lungs of patients with cystic fibrosis are highlighted by large gray arrows. Therapeutic classes that have been and/or are being investigated for the chronic management of cystic fibrosis lung disease are shown in light gray boxes. Despite different mechanisms of action, all share the goal of reducing lung disease damage caused by the interplay of obstruction, infection, and inflammation. Reprinted with permission from VanDevanter DR, Konstan MW: Outcome measures for clinical trials assessing treatment of cystic fibrosis lung disease. *Clinical Investigation *2012; 2(2):163-175 [152].

Organ systems pathologies persist and evolve after birth. More than 90% of infants with CF lose all exocrine pancreatic function and experience a lifetime of pancreatic insufficiency [6]. The risk of CF-related diabetes mellitus increases with age, occurring in more than a quarter of individuals 25 years of age and older [1]. Gastrointestinal complications continue, with individuals experiencing fat and fat-soluble vitamin malabsorption and associated steatorrhea, poor growth, increased risk of gallstones and hepatobiliary disease [6]. Interestingly, the organ system that currently accounts for the greatest percentage of premature deaths from CF, the respiratory system, appears essentially normal at birth. Of course, respiration only begins at birth, and complications arising from reduced CFTR activity in the sinuses and respiratory tract can become apparent remarkably soon after [9-16]. A summary of the pathophysiology in the lung is shown in Figure 1. Reduced CFTR activity results in volume depletion of the lung apical surface liquid (ASL), leading to increased adhesiveness and cohesiveness of airway phlegm, effectively plugging the small airways [17]. This obstruction, and associated neutrophilic inflammation, can be identified as air trapping and bronchial wall thickening using high-resolution computerized tomography [14,15]. Bacterial opportunists enter the upper and lower respiratory tract by inhalation or aspiration, from where it is not possible to clear them; their growth and expansion leads to local inflammation [9-12]. Very early, this triad of chronic obstruction, infection and inflammation sets in motion a lifelong degradation of lung anatomy and function [6,16,18,19], ultimately contributing to the untimely deaths of persons with CF; respiratory failure accounts for >80% of mortality in CF [1].

## The relationship of chronic cystic fibrosis therapies to underlying pathophysiology

Chronic management of CF today is focused primarily on the mitigation of downstream pathologies that accumulate due to reduced CFTR activity in the digestive and respiratory systems. Pancreatic insufficiency and poor absorption of fat and fat-soluble vitamins are reasonably well-managed by pancreatic enzyme supplementation at every meal, consumption of a high fat and high calorie diet, and vitamin supplementation [20]. Careful attention to patient nutrition has produced dramatic improvements in nutritional status and has contributed to improved overall health for all ages of patients with CF [1]. Gastrointestinal tract obstruction, experienced by about 25% of adults with CF in their lifetime, is treated both pharmacologically and surgically [6]. Chronic pulmonary therapies target the movement of tenacious phlegm out of the respiratory tract, suppression of chronic bacterial infection and reduction of chronic local inflammation. Surgical intervention is also an important component of respiratory tract management, being common for removal of sinus polyps [21], in rare cases to arrest hemoptysis [22] and, for those with end-stage bronchiectasis, bilateral lung transplantation [23].

A primary complication of CF is the accumulation of airway phlegm that contains almost no intact mucin and consists predominantly of bacteria, inflammatory cells, polymeric DNA and F-actin, with characteristics more similar to pus than mucus. A variety of chest physiotherapies are recommended for improving clearance of airway phlegm [24]. These therapies can be combined with medications that might alter the airway phlegm, making them easier to clear from the airways.

Growth and expansion of inhaled or aspirated bacterial opportunists within the airway of patients with CF are treated with antibiotics in four distinct modalities: chronic prophylaxis to avoid (specific) bacterial infection; conversion to culture negativity upon detection of new (specific) bacterial opportunists; palliation of acutely elevated signs and symptoms of infection; and chronic suppression of established infections. CF antibiotic treatment modalities have evolved over years of trial and error, some without strong objective evidence of clinical benefit, and have features that range from the uncommon to the heretical when compared with the antibiotic management of systemic bacterial infections. Management of increased respiratory signs and symptoms (loosely defined as pulmonary exacerbations) routinely includes antibiotic treatment [25]. Although exacerbations treated with intravenous antibiotics have tended to receive the most attention from epidemiologists [26-32] and drug developers [33-37], they appear to constitute a minority of acute, antibiotic-treated events, particularly in younger patients [38]. Surprisingly, although tens of thousands of these events occur annually and they have been associated with decreased quality of life [39-41], accelerated lung function decline [27,28] and increased mortality risk [42-45], little objective evidence of the benefit of antibiotic treatment is available and important questions as to optimal antibiotic treatment of exacerbations remain [46].

Local airway inflammation is accepted as a critical driver of lung disease progression and death [6], but therapies directly targeting local inflammation have been slow to achieve widespread use [47], in part because of the concern over treatment-associated adverse events.

## Interventions more directly targeting the cystic fibrosis defect

CF disease sequelae arise from reduced CFTR function in disparate tissues, and it follows that the most comprehensive and effective treatment of CF would be reconstitution or supplementation of CFTR activity prior to the establishment of irreversible functional and structural damage. Substantial progress has been made toward this very approach, but with important limitations. As noted above, multiple pathophysiologic consequences of reduced CFTR arise *in utero*, necessitating downstream management of subsequent disease sequelae even in very young infants with CF. For example, fibrosis of the exocrine pancreas, when it occurs, is likely nearly complete at birth and reconstitution of CFTR function in the pancreas of neonates is unlikely to lead to pancreatic sufficiency later in life. However, the observation that the respiratory tract is, by comparison, relatively unaffected at birth suggests that reconstitution or supplementation of CFTR activity in the airway in infants with CF may have a dramatic effect on lung disease progression.

## Current and future therapies

Now that we have outlined the general approaches to the various aspects of the pathophysiology of CF lung disease, we can review the details of specific therapies that are currently in use, or are in active stages of development, and could be realized as approved therapies in the near future. These are addressed in the general order of the pathophysiologic mechanisms (Figure 1).

### CFTR correction

Shortly after identification of the *CFTR *gene, there was particular interest in and hope that insertion of a normal copy of the gene (that is, gene therapy) would restore CFTR function in persons with CF [5]. Gene therapy has long been promised as the pathway to a cure for CF and an assumed advantage of gene therapy for CF was that the airways should present a relatively easy target to reach. However, much has been learned from gene therapy trials and the airways present many barriers to successful gene therapy, more so than many other organs. The reader is referred to a recent review of the status of CF gene therapy investigations [48], but what has been demonstrated is that the principle of transfer of the *CFTR *gene is possible and that a partial correction of the basic defect in the airways of patients with CF can be achieved without major safety issues. What has yet to be demonstrated is that *CFTR *gene transfer to the lung will result in a clinical benefit and whether gene transfer and/or expression can be successfully repeated over what would be decades of treatment. The UK Cystic Fibrosis Gene Therapy Consortium is planning to initiate a multi-dose clinical trial of gene therapy in 2012, which will be the first opportunity to find out if gene therapy can result in clinical benefit.

### CFTR modulation

Although we cannot currently supplement mutant *CFTR *alleles with a normal copy by gene therapy, small molecules have been identified that can modulate mutant *CFTR *protein such that its function may be closer to normalcy. There are more than 1,800 CF gene mutations that have been identified, but not all are known to cause disease, allowing for some patients to have minimal symptoms yet believed to have CF. For those mutations known to cause complications of CF, we categorize them based upon the major presumed defect (that is, synthesis, processing, regulation and channel conductance) [49,50], but these categories are not highly specific and there is considerable overlap for some mutations (that is, they could be sorted into more than one category). The general approach to evaluating CFTR-modulating drugs is to assess their ability to either increase the quantity of CFTR at the cell surface and/or increase the function of CFTR.

The first approved CFTR modulator, ivacaftor, is indicated for patients with CF with a specific mutation, G551D. This mutation results in a reduced probability of opening of the CFTR channel (an altered gating mechanism); there is a sufficient quantity of CFTR but there is impaired function of the channel [51]. Ivacaftor potentiates the CFTR channel by increasing the probability of channel opening [51]. A clinical trial in patients with CF with at least one copy of G551D demonstrated proof of this concept with a decrease in mean sweat chloride from approximately 100 mEq/L to approximately 51 mEq/L [52]. More importantly, there were remarkable clinical benefits with an increase in forced expiratory volume in one second by 10.6% of predicted value, a reduction in risk of pulmonary exacerbations by 55%, an increase in weight and an improvement in quality of life [52]. Similar results have been demonstrated in pediatric patients and ivacaftor has been approved by the Food and Drug Administration (FDA) for patients aged six years and above. There are important questions that remain to be answered, including whether the drug be used in newly diagnosed infants, will the drug work in other mutations (especially gating mutations), and will the drug have an effect on other manifestations of disease (for example, sinus disease, gastrointestinal absorption, reduction in inflammation in the airways)?

Studies that address the first question should easily be able to demonstrate an effect on sweat chloride and any new safety issues but other clinical benefits will be more challenging; is it possible that sweat chloride could serve as an acceptable surrogate clinical endpoint? As to the second question, we know that ivacaftor will not be an effective therapy alone in patients homozygous for F508del *CFTR *mutations [53]. This is not an unexpected finding as the F508del mutation results in abnormal processing of the CFTR protein such that there is a marked reduction in the quantity of CFTR at the cell surface [54,55]. However, it is likely that ivacaftor will potentiate F508del protein [56], so if the quantity of F508del protein could be increased at the cell surface by a small molecule, there is the possibility that ivacaftor could be used in combination with it to increase CFTR activity. There are promising agents that may allow for correction of the CFTR processing defect seen with F508del mutations, including VX-809 [57] and VX-661 [58]. These are currently under investigation in combination with ivacaftor in patients with F508del mutations [59,60]. The last question is being addressed in an observational study of ivacaftor in patients with G551D (the GOAL study) sponsored by the Cystic Fibrosis Foundation [61]. This provides an opportunity to understand the biology of CFTR modulation, including the direct and indirect effects of activating CFTR-dependent anion secretion by collecting specimens and clinical data on a large number of patients both before and after they begin treatment with ivacaftor.

A specific type of mutation called a nonsense (premature stop codon) mutation also results in a reduction in the quantity of CFTR protein at the cell surface. These mutations, affecting approximately 10% of patients with CF, prematurely halt translation of mRNA to protein, resulting in incomplete CFTR proteins [62]. The strategy necessary to address this problem would require 'reading through' the mutation to the normal stop codon. Ataluren is an orally delivered investigational drug that has the potential to overcome the effects of the nonsense mutation and has been shown to induce the production of full-length functional CFTR protein at epithelial cell surfaces in mice [63] and humans [64]. Short-term studies of ataluren in pediatric [64] and adult [65] patients have shown treatment-induced improvements in CFTR-mediated chloride transport in respiratory epithelium. A Phase III clinical trial is currently under investigation in adult and pediatric patients [66].

### Alternate channels

CFTR is not the only channel on the epithelial cell surface responsible for the maintenance of the airway surface liquid. There are other chloride channels including a calcium-dependent chloride channel [67] and the P2Y receptor activated by ATP [68]. Early studies of uridine-5 triphosphate, an analog of ATP, found its short half-life limited its clinical value [69]. A subsequent analog, denufosol, showed greater stability and early trials held great promise. A Phase III study (the Transport of Ions to Generate Epithelial Rehydration study (TIGER1)) demonstrated lung function improvement [70]; however, a second placebo-controlled study (TIGER2) did not duplicate these results and further development for CF was discontinued [71]. Denufosol was observed to have a relatively short half-life (17 minutes) in a pharmacokinetic sub-study, much shorter than had been predicted from *in vitro *and *ex vivo *studies [72], and it may be that study failure was due to simple pharmacokinetics. It is also possible that agents such as denufosol may be capable of changing the rate of lung function decline without necessarily improving lung function, in which case the choice of lung function improvement as the primary efficacy endpoint in the denufosol studies was problematic.

Lancovutide increases intracellular calcium level and activates an alternative chloride channel [73]. A proof-of-concept clinical study showed promise with increased chloride conductance, as measured by nasal potential difference, when applied topically to nasal epithelium in patients with CF [74], and a small, single-center clinical trial in 24 patients with CF found that it was safe and benefitted pulmonary function [75]. However, there has been no further development of this drug in clinical trials.

One of the roles of CFTR is to inhibit sodium absorption and its absence causes excessive sodium (and water) absorption through an epithelial sodium channel [76]. An alternative approach to CFTR treatment could be to inhibit sodium absorption through the epithelial sodium channel. Aerosolized amiloride (an epithelial sodium channel blocker with a short half-life) not only had no clinical benefit, it showed a trend toward poorer lung function in the treated patients [77]. There may yet be other channels identified as more relevant in CF lung disease, or agents with a longer half-life may be a better option.

### Hydrators

Another proposed approach to restoring airway surface liquid is the direct instillation of salt and water. This has been demonstrated to increase the volume of airway surface liquid in cultured CF airway epithelia [78] and it has been suggested that inhalation of hypertonic saline can improve mucociliary clearance *in vivo *[79]. In this latter study, the benefit occurred in the first 60 minutes [79], suggesting either that the increase in mucociliary clearance is a short-lived effect or it is the result of another mechanism, such as transiently increasing the volume of ASL, unbinding secretions from the airway surface and inducing cough. In any case, aerosolized hypertonic saline has demonstrated other clinical benefits. Twice daily inhalation of hypertonic (7% to 8%) saline has been shown to improve ventilation inhomogeneity in patients with CF with 'normal' spirometry [80]; reduce sputum markers of inflammation [81]; reduce the risk of pulmonary exacerbation [36,82]; and modestly improve pulmonary function [36,83]. Dose frequency [78] and concentration [84] may affect the magnitude of the inhaled hypertonic saline response. Recently, a large randomized placebo-controlled multicenter 48-week study of inhaled hypertonic saline in children with CF under six years of age failed to demonstrate a treatment-related reduction in the rate of pulmonary exacerbation [85]. There has yet to be a definitive demonstration that inhalation of lower saline concentrations (commonly prescribed to increase tolerance and adherence) provide clinical benefit [84].

The challenges of hypertonic saline include poor tolerance because of increased cough and bronchospasm [36] and the time it takes for aerosolization, adding to the patient's treatment burden. As stated earlier, the benefit of aerosolized hypertonic saline occurs early and could be related to the short time that the drug can affect the phlegm because of rapid absorption. An alternative approach is to deliver an osmotic agent to the airways that could draw water into the airway surface liquid and have a longer presence for which it to be active. Recently, a dry powder formulation of mannitol for inhalation has been developed that presumably shares the inhaled hypertonic saline mechanism(s), and it has been studied in both CF and non-CF bronchiectasis. Similar to the data for hypertonic saline, the improvement in airway clearance also appears to occur in the first 45 minutes, after which the rate of clearance appears to be the same in all doses tested [86]. Clinical trials of inhaled mannitol resulted in improved lung function and a trend to reduced rates of pulmonary exacerbations; these results seemed to be sustained in an open-label extension of the study [87]. The drug is currently under review by the FDA. As with hypertonic saline, a substantial minority of patients with CF is intolerant to this therapy, with cough being a frequently observed adverse event [87].

Xylitol is another sugar with low transepithelial permeability that may function as an osmolar agent similar to mannitol. Early studies have demonstrated safety in mice, healthy volunteers and stable patients with CF when administered over a single day [88]. Ongoing clinical trials will investigate the safety and efficacy of inhaled xylitol compared with hypertonic saline [89].

### Airway clearance therapies

Mechanical clearance of phlegm from the airways is one of the primary therapies for CF airways disease. Because there is impaired mucociliary clearance, the patient is dependent upon cough clearance to rid the airways of the infectious and inflammatory material accumulating in the airways. There are several accepted methods of airway clearance, and guidelines published by the Cystic Fibrosis Foundation Pulmonary Guidelines Committee offer several specific recommendations [24], which can be summarized as follows:

1. some form of airway clearance therapy should be performed in all patients on a routine basis;

2. no single method of airway clearance therapy has been proven superior to any other, but this does not mean they are equivalent in all patients;

3. because of the previous recommendation, there should be a strategy of determining which therapy works best for each patient;

4. exercise is a great addition to the airway clearance regimen.

It is unlikely that there will be any new trials that will compare these therapies (or new versions) to determine superiority. The best strategy is to use an algorithm by which all therapies are introduced to patients at an appropriate age to determine which will be used and which will be the most effective [90].

### Dornase alfa

Medications have been used to alter the properties of airway phlegm to make it easier to clear from the airways. Both hypertonic saline and mannitol have been demonstrated to alter the properties of sputum and this may be an important component of their clinical benefit [86,91]. Recombinant human DNase (dornase alfa) has been developed to cleave high molecular weight DNA released by dead neutrophils that contributes to the tenacity of airway phlegm [92]. Daily inhalation of aerosolized dornase alfa can reverse early air trapping [93] and ventilation inhomogeneity [94]; decrease sputum markers of local inflammation [92,95]; reduce pulmonary exacerbation risk [33,35]; improve CF pulmonary function [33,35]; slow the rate of at which pulmonary function is lost [96]; and improve survival [97]. It is generally well-tolerated and has become a mainstay in the recommended treatments to maintain lung health [98].

### Aerosolized antibiotics

As stated earlier, there are several situations in which antibiotics are used to treat CF airways disease. Chronic prophylaxis primarily targets acquisition of *Staphylococcus aureus *in infants by use of oral flucloxacillin [99], but is used only regionally (primarily in the UK and Europe) based upon local experience and tradition, the differential weighting of apparently conflicting study results [99-101] and drug availability. Newly detected *Pseudomonas aeruginosa *from throat cultures has been treated with inhaled antipseudomonal antibiotics [102-105] with or without concomitant oral ciprofloxacin and intravenous antipseudomonal antibiotics. Although these interventions are routinely capable of converting >80% of patients to *P. aeruginosa *culture negativity [104,106-109], the ultimate clinical benefit of delaying chronic *P. aeruginosa *infection with this approach has not been robustly demonstrated.

Ironically, the potentially most heretical use of antibiotics in CF, the chronic suppression of bacterial opportunists in the airway without their eradication, is the one with the greatest empirical evidence for clinical benefit. Chronic or chronic intermittent administration of the inhaled antipseudomonal antibiotics colistimethate, tobramycin and aztreonam has been associated with improved quality of life [110,111], decreased risk of exacerbation [34,112], improved pulmonary function [34,111], and decreased mortality [97]. This practice, which has expanded substantially in recent decades [47], was predated by scheduled, periodic treatment of patients with intravenous antibiotics [113,114].

### Anti-inflammatory medications

The inflammatory response in the airways of patients with CF is excessive and it is thought that the inflammation overwhelms the natural protective mechanisms of the airway [115]. It is the excessive and persistent inflammation that is likely to be the main cause of destruction of the airways over time, so it is logical that anti-inflammatory therapy could provide benefit to the progression of CF airways disease.

Chronic administration of oral prednisone has been shown to improve pulmonary function in children with CF [116] but also to result in significant growth impairment [117] in a population with preexisting growth challenges. Inhaled corticosteroids may reduce the potential for adverse effects, but the evidence for the benefit of chronic inhalation of corticosteroids is conflicting [98,118-120]. There has never been a randomized controlled trial describing the treatment benefit of inhaled corticosteroids, although a controlled study has demonstrated that withdrawal of the inhaled corticosteroid fluticasone was not associated with an increased rate of exacerbation or reduced pulmonary function during a six-month post withdrawal period [118]. However, two retrospective CF registry analyses have independently shown an association between chronic inhaled corticosteroid use and a reduced rate of lung function decline over a period of years [119,120], again suggesting that short-term measures of respiratory health may not be perfect surrogates for longer-term disease progression. Current US chronic respiratory therapy guidelines recommend against the use of inhaled corticosteroids in patients without asthma [98], although their use remains extremely common [47], perhaps in part because of the very low treatment burden associated with the administration of drug via a metered-dose inhaler.

As an alternative, nonsteroidal anti-inflammatory drugs have been studied as a treatment of CF lung disease. Ibuprofen, when taken in high doses, inhibits the migration, adherence and aggregation of neutrophils [121-123]. Despite prospective and retrospective demonstration that chronic administration of high dose ibuprofen reduces the rates of pulmonary function decline [124-126], less than 10% of eligible patients in the US receive this therapy [1,47] due to concerns for rare, but very serious, gastric bleeding associated with the treatment [124].

Macrolide antibiotics are currently recommended as a chronic therapy for patients with CF to improve lung function and reduce exacerbations [98], despite some degree of uncertainty as to the exact mechanism(s) by which macrolides exert their clinical effect. Although azithromycin, the macrolide most commonly prescribed to patients with CF, is marketed as an antibiotic, there is substantial evidence that a reduction in airway inflammation plays a key role in its efficacy in CF. The clinical evidence of a macrolide-derived anti-inflammatory effect includes a decrease in the number of neutrophils present at the site of infection [127-130] and a reduction in pro-inflammatory cytokines that recruit more neutrophils [131,132]. EM703, a macrolide variant with essentially no antimicrobial activity [133], retains strong immunomodulatory properties and has been shown to protect against chemically-induced pulmonary fibrosis in an animal model [134]. Generally, macrolides such as azithromycin are not considered to be effective against *Pseudomonas *using traditional *in vitro *susceptibility testing for CF bacterial isolates, although reports of clinical benefit have been derived from large studies in persons with CF and chronic *P. aeruginosa *infection [135,136]. However, *in vitro *studies have suggested that the clinical efficacy of azithromycin may result directly from a cidal activity against *Pseudomonas *[137,138] or indirectly by inhibition of bacterial biofilm production [139,140]. Patients with CF receiving chronic azithromycin have been shown to culture strains of *S. aureus *and *Haemophilus influenzae *with progressively reduced macrolide sensitivities over time, indicative of an antibiotic effect, although not one directed against *Pseudomonas *[141]. The question of whether the macrolide benefit in CF results from either direct suppression of inflammation or from an antimicrobial effect, or both, is of more than academic importance. Presumably, individuals are less likely to become refractory to an anti-inflammatory effect with chronic exposure, while the same may not be true for chronic antibiotic exposure.

As stated earlier, the neutrophil-dominated inflammation in the airways overwhelms the natural protection, notably the neutrophil elastase alpha1-antitrypsin (A1AT). When given in aerosol form to 12 patients with CF, A1AT suppressed neutrophil elastase in the respiratory ASL and reversed the inhibitory effect of CF ASL on *Pseudomonas*-killing by neutrophils [142]. Early studies of A1AT treatment have demonstrated a decrease in inflammatory parameters after treatment [142,143]. The challenge to this therapy is the ability to deliver an effective dose to the airways. New technologies of aerosol devices may make this therapy more viable.

Another observation in CF airways disease is that there is a reduction in glutathione, an important component of airway defense, in the lower airways as measured in bronchoalveolar lavage fluid [144]. It has been suggested that replacement of glutathione directly, or indirectly be replacing sulfhydryl groups through administration of N-acetyl cysteine (NAC) might reduce the inflammatory response. A pilot study of inhaled glutathione in 19 patients with CF suggested a trend towards improved inflammatory outcomes [145]. Early trials suggested that high dose NAC might also reduce the inflammatory state, but a single-center study failed to demonstrate a change in clinical or inflammatory parameters in the bronchoalveolar lavage fluid of patients [146]. Nonetheless, extracellular glutathione in induced sputum tended to increase on high-dose NAC, suggesting there was an effect on increasing available glutathione. Whether enhancing airway glutathione levels by either approach will improve CF lung health remains to be demonstrated.

### Lung transplantation

For those patients who progress to an advanced severity, lung transplantation may be an option. In carefully selected patients, lung transplantation has demonstrated great success with a one-year survival of approximately 90%, but the five-year survival is only 50%, most likely because of the development of chronic rejection as bronchiolitis obliterans [147]. The etiology of chronic rejection remains debated but a prime strategy of treating it, or trying to prevent it, is chronic administration of anti-rejection medications, such as cyclosporine A and tacrolimus [148]. A principal adverse event associated with long-term immunosuppression is chronic renal insufficiency, a complication that has led to the study of inhaled cyclosporine A (originally studied as a rescue medication for acute transplant rejection [149]) as a topical alternative for long-term immunosuppression to reduce systemic exposure. A small single-center randomized controlled clinical study suggested that treatment of lung transplant recipients with inhaled cyclosporine A beginning shortly after transplant reduced chronic rejection and improved survival while not increasing the risk of infection [149]. A large multicenter controlled clinical trial of inhaled cyclosporine (NCT00755781) has apparently completed enrollment, but no results from this Phase III study have been released.

## Conclusions

The last two decades have seen a remarkable addition of effective medications and therapies to the regimen for treating CF lung disease. These therapies have improved the overall health of patients with CF and they are clearly part of the reason that expected survival has increased. However, these therapies do not offer a cure and they mainly treat downstream complications of the pathophysiology of CF lung disease, meaning that patients continue to suffer the morbidity associated with chronic airways infection and predicted survival still lags well below what is normal. In addition, these therapies add to a considerable treatment burden and so are also associated with poor adherence.

There are two main areas we need to focus on for there to be further improvement. First, we must develop new, more effective medications. Ideally, these would be directed at earlier events in the pathophysiology of disease, such as seen with ivacaftor, and perhaps earlier use of such medications would prevent, or at least delay, the development of downstream events such as chronic infection of the airways. Another approach would be to change the formulation of current medications to reduce the treatment burden, and hopefully increase adherence to the medication. An example is the development of a dry powder to replace an aerosol medication, such as tobramycin inhalation powder, which has been shown to be equivalent to tobramycin inhalation solution in terms of efficacy, but is associated with a markedly reduced treatment time and greater patient satisfaction [150]. There is also a need for the development of additional agents within the current classes to enhance the treatment options should current treatments seem to be less effective. An example is the addition of a class of inhaled antibiotics. Currently we have an aminoglycoside and a beta-lactam antibiotic approved for aerosol use in CF, and a fluoroquinolone, which is in the late stages of development [151], would add to the treatment options.

Second, there must be optimization of the currently available therapies. All of the existing therapies were developed compared to placebo and we have little information about which combination of drugs is most effective. We also do not know if there is a more effective order in which to use the medications. Comparative effectiveness research may be an effective method of identifying the optimal treatment strategy for patients, perhaps allowing elimination of some drugs for some patients, again reducing the treatment burden. Not discussed here but also very important is the development of new devices that are intended to improve the delivery of drug to the lower airways; these devices are more efficient and so can deliver the needed dose in a much smaller volume and, therefore, a much shorter time.

There are medications currently up for review by the FDA, including mannitol and tobramycin inhalation powder and another (levofloxacin inhalation solution) that is very near completion of Phase III trials, meaning the CF armamentarium could have as many as three new weapons in the very near future. These will be important options for our patients. However, it is the *CFTR *modulators that have generated the most excitement. Although they are not a cure, they represent our first successes at treating early events in the pathophysiology of CF lung disease, meaning they have real potential for being truly disease modifying. Although the approval of ivacaftor offers treatment to a minority of patients (<4%), the demonstration that this strategy can work offers great hope to the CF community. New medications will have to be developed for each type of mutation and having two new medications in active investigation for F508del, by far the most common *CFTR *mutation, brings us that much closer to making a huge difference in the lives of our patients.

## Competing interests

The authors declare the following competing interests. PAF declares grant support from entities with interests in CF including Mpex Pharmaceuticals, Inc., Gilead Sciences, Inc., Bayer Healthcare AG, Novartis, Vertex Pharmaceuticals, Inc., Pharmaxis Limited., and Boehringer Ingelheim Pharmaceuticals. DRV declares consultant work for KaloBios, Genentech, Inc., Mpex Pharmaceuticas, Inc., Baxter HealthCare, Cystic Fibrosis Foundation, Inspire, NanoBio, DKB medicine, Mylan, Pulmatrix, and KMK.

## Authors' contributions

PAF and DRD contributed equally to the drafting and revising of the manuscript. Both have given final approval of the version to be published.

## Pre-publication history

The pre-publication history for this paper can be accessed here:

http://www.biomedcentral.com/1741-7015/10/88/prepub
